# Surface-induced dissociation of protein complexes on a cyclic ion mobility spectrometer†

**DOI:** 10.1039/d1an01407b

**Published:** 2021-10-11

**Authors:** Dalton T. Snyder, Benjamin J. Jones, Yu-Fu Lin, Dale A. Cooper-Shepherd, Darren Hewitt, Jason Wildgoose, Jeffery M. Brown, James I. Langridge, Vicki H. Wysocki

**Affiliations:** Resource for Native MS Guided Structural Biology, The Ohio State University Columbus OH USA 43210; Department of Chemistry and Biochemistry, The Ohio State University Columbus OH USA 43210 wysocki.11@osu.edu; Waters Corporation, Stamford Avenue Altrincham Road Wilmslow SK9 4AX UK

## Abstract

We describe the implementation of a simple three-electrode surface-induced dissociation (SID) cell on a cyclic ion mobility spectrometer (cIMS) and demonstrate the utility of multipass mobility separations for resolving multiple conformations of protein complexes generated during collision-induced and surface-induced unfolding (CIU & SIU) experiments. In addition to CIU and SIU, SID of protein complexes is readily accomplished within the native instrument software and with no additional external power supplies by entering a single SID collision energy, a simplification in user experience compared to prior implementations. A set of cyclic homomeric protein complexes and a heterohexamer with known CID and SID behavior were analyzed to investigate mass and mobility resolution improvements, the latter of which improved by 20–50% (median: 33%) compared to a linear travelling wave device. Multiple passes of intact complexes, or their SID fragments, increased the mobility resolution by an average of 15% per pass, with the racetrack effect being observed after ∼3 or 4 passes, depending on the drift time spread of the analytes. Even with modest improvements to apparent mobility resolving power, multipass experiments were particularly useful for separating conformations produced from CIU and SIU experiments. We illustrate several examples where either (1) multipass experiments revealed multiple overlapping conformations previously unobserved or obscured due to limited mobility resolution, or (2) CIU or SIU conformations that appeared ‘native’ in a single pass experiment were actually slightly compacted or expanded, with the change only being measurable through multipass experiments. The work conducted here, the first utilization of multipass cyclic ion mobility for CIU, SIU, and SID of protein assemblies and a demonstration of a wholly integrated SIU/SID workflow, paves the way for widespread adoption of SID technology for native mass spectrometry and also improves our understanding of gas-phase protein complex CIU and SIU conformationomes.

## Abstract 2

Characterization of protein assemblies and amyloid aggregates by CIU, CID, SIU, and SID on a cyclic ion mobility spectrometer.

## Graphical abstract

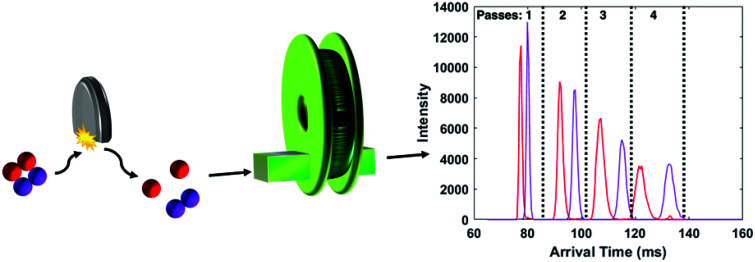

## Introduction

Native mass spectrometry (nMS) is a growing field that provides information complementary to that acquired with traditional structural biology methods such as X-ray crystallography and nuclear magnetic resonance.^1,2^ nMS is a powerful technique that allows the study of proteins and protein complexes in the gas phase by kinetic trapping of solution structures during the electrospray process,^3^ enabling the determination of stoichiometry, ligand binding, oligomeric state, and, when coupled with ion mobility, collision cross section measurements (which can be compared to structures determined by other structural biology tools or computational structure predictions).^4–6^

Coupling native mass spectrometry with activation methods such as collision-induced dissociation (CID), electron capture dissociation (ECD), electron transfer dissociation (ETD), and ultraviolet photodissociation (UVPD) allows structure and sequence information to be determined.^7–10^ Surface-induced dissociation (SID) allows ligand binding and subunit connectivity information to be obtained as well.^11–13^ SID typically produces substructure fragments that are reflective of the native solution structure of the protein complex of interest. Moreoever, these fragments remain compact, while CID produces highly charged monomer (that is typically extended) and the complementary (*N* − 1)mer.^14,15^ SID of native protein complexes to date has been performed by expert groups on custom modified instruments; the complexity and size of prior generations of SID devices has hindered widespread adoption^15–19^ but the unique and informative fragmentation patterns obtained by SID motivated simplification of the ion optics,^20–22^ dissemination of the technology to beta testers,^17,23^ and collaboration with instrument companies.

Ion mobility is a gas-phase separation technique that separates ions by size, shape, and charge in the presence of a background gas. This separation occurs on the millisecond timescale which couples well with a fast mass analyzer such as time-of-flight (TOF) operating on a microsecond timescale.^24^ Traveling wave ion mobility was commercialized by Waters Corporation (Manchester, UK) with the Synapt HDMS platform as initially described by Giles *et al.* and allowed for the study of gas-phase conformations of protein complexes.^25,26^ Improvements to traveling wave ion mobility, namely the installation of a helium-filled cell prior to the main nitrogen-filled IMS cell, allow for increased mobility pressures with minimal ion loss, increasing achievable ion mobility resolution.^27^ Increased ion mobility resolution was obtained on the SELECT SERIES Cyclic Ion Mobility Spectrometer (cIMS) by a combination of increased ion mobility path length (98 cm *vs.* 25 cm in the Synapt G2 and G2-S) and the ability to extend separation length by allowing ions to make multiple passes through the cyclic ion mobility device,^28^ inspired by Clemmer's initial studies.^29^ A miniaturized surface-induced dissociation device developed in the Wysocki lab has enabled the installation of the same device design in multiple instrument platforms with minimal adaptation.^22^ In the Synapt and cIMS platforms, the latest generation (‘Gen 3’) SID device replaces the Dynamic Range Enhancement (DRE) lens and through additional hardware and software upgrades represents a wholly integrated SID implementation on the cIMS.

Surface-induced dissociation of protein complexes often produces overlapping fragments in *m*/*z* that can be resolved by the use of ion mobility or isotopic resolution.^13,22^ The increased ion mobility and mass resolution on the cIMS allow for the analysis of more complex systems while maintaining ion mobility separation or isotopic resolution for smaller protein complexes.^30^ Increased mobility path length additionally is likely to benefit collision-induced unfolding or surface-induced unfolding experiments wherein a variety of restructured precursor ions (collapsed but unfolded, compact but flexible) can be produced even at a single collision energy.^18,31,32^ The utility of high-resolution ion mobility separations is demonstrated here for several homomeric and heteromeric protein complexes that benefit from the 4x increased drift length and analytical flexibility of the cIMS platform. The increase in ion mobility resolution for single and multipass experiments is quantified in this work and further utilized to improve our understanding of protein conformations produced *via* CIU and SIU experiments.

## Experimental

### Sample preparation

Recombinant streptavidin, cholera toxin B, phosphorylase B from rabbit muscle, and alcohol dehydrogenase (from *saccharomyces cerevisiae*) protein standards received as lyophilized powers (from Sigma Aldrich) were reconstituted in ultrapure water generated on site (Sartorius Arium Pro, Göttingen, Germany) and aliquoted and stored at −20° C until use. Recombinant C-reactive protein was purchased from EMD Millipore. Neutravidin from egg white was purchased from Thermo Scientific. HFQ65 was obtained from the Woodson group (Johns Hopkins University), *trp* RNA binding attenuation protein (TRAP) was obtained from the Foster group (The Ohio State University) and the Gollnick group (State University of NY, Buffalo), and toyocamycin nitrile hydratase (TNH) was received from the Bandarian group (The University of Utah). Samples were buffer exchanged into 200 mM ammonium acetate (99.99%, Millipore Sigma) using Micro Bio-Spin P6 spin columns (Bio-Rad, Hercules, CA, USA). Charge-reduced samples were prepared by adding triethylammonium acetate (TEAA) (Millipore Sigma) or ethylenediamine diacetate (EDDA) (Sigma), giving final concentrations of 160 mM ammonium acetate and 40 mM TEAA or EDDA. Samples were diluted to 5–10 μM protein complex before analysis. Trimers derived from Aβ_17–36_, discussed later, were synthesized by Nowick laboratory (at University of California, Irvine)^33^ and initially dissolved in ultrapure water and flash frozen with liquid nitrogen at 200 μM stock concentration. A working solution of 40 μM was obtained by diluting with 400 mM ammonium acetate.

### Ionization

Gas-phase ions were produced by nanoelectrospray ionization using pulled capillaries (thin wall borosilicate glass, 1.00 mm outer diameter, 0.78 mm inner diameter; Sutter Instruments) with ∼2 μm tip diameters made in-house by a Sutter Instruments P-97 tip puller (Novato, CA). A platinum wire (0.25 mm inner diameter, Thermo Fisher Scientific) inserted into the capillary served as a means of applying voltage (∼1 kV) to generate the electrospray.

### Instrumentation

**Fig. 1:**
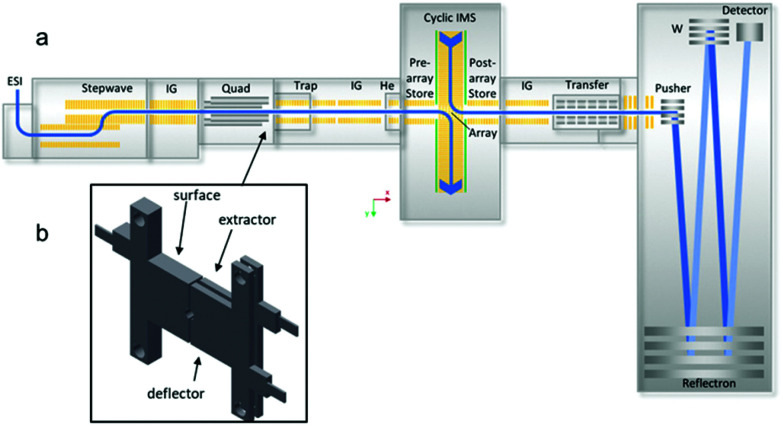
Schematic of the cyclic IMS (cIMS) modified in this work: (a) instrument diagram showing location of SID cell after the quadrupole and prior to the trap collision cell; (b) 3-lens SID cell (3.6 mm length along the ion optical axis). The SID cell is fully controlled in the commercial instrument software (Quartz) and does not require any external power supplies. Adapted from K. Giles, J. Ujma, J.Wildgoose, S. Pringle, K. Richardson, D. Langridge and M. Green, *Anal. Chem.*, 2019, **91**, 8564–8573.

A SELECT SERIES Cyclic IMS (cIMS) Q-cIMS-TOF^28^ and a Synapt G2 HDMS Q-IM-TOF^34^ ion mobility mass spectrometer from Waters Corporation (Wilmslow, UK) were used for these studies. Fig. 1a is a schematic of the cIMS.^35^ The instrument consists of a nanoESI ion source connected to a stepwave ion guide to direct ions through the intermediate pressure source (∼2.2 torr) while rejecting neutrals and a second ion guide located in the second differential pressure region. The ions can then be mass-selected in the quadrupole (up to *m*/*z* 32 000) and subjected to activating collisions, either *via* SID or CID. As shown in Fig. 1b the surface-induced dissociation cell is located between the selection quadrupole and the Trap stacked ring ion guide, replacing the commercial Dynamic Range Enhancement (DRE) lens used for programmable attenuation of the ion beam. Notably, the DRE lens design is identical in the Synapt G1, G2, G2-S, and Select Series cIMS platforms – aside from an evolution of the inner diameter from 2 mm on G1 and G2 to 2.5 mm on the others – and so the SID hardware used in this work is nearly universal on these platforms. The ions are then collected in the trap collision cell and pulsed into the ion mobility cell on the cIMS and the G2. The path length of the G2 is 25.4 cm,^34,36^ while the CIMS single-pass length is 98 cm, theoretically providing a resolution increase of 2 (other factors notwithstanding). After passing through the IMS separation devices on each platform, the ions are directed toward either (G2) a ‘transfer’ stacked ring ion guide (collision cell) or (cIMS) a post-IMS stacked ring ion guide followed by a segmented quadrupole with an axial DC gradient, in which the ions’ mobility separation is maintained. Lastly, the mobility-separated ions are mass analyzed by the oa(orthogonal acceleration)-TOF. The cIMS has a longer TOF analyzer featuring improved pumping and improved resolution.

Both instruments are operated with nitrogen serving as the cooling gas in the trap and transfer regions. Source conditions on both instruments have been tuned to maximize ion transmission while minimizing ion activation to avoid possible restructuring. Settings in the ion mobility cells were as follows: (cIMS) 120 mL min^−1^ helium cell gas flow rate; (cIMS) 40 mL min^−1^ or (G2) 60 mL min^−1^ gas flow rate into the ion mobility cell; wave height of 25 V; wave velocity of 375 m s^−1^. Mass and mobility data were processed in MassLynx and Driftscope (Waters Corporation). Mass spectra were generally smoothed 3 times with a smoothing window of 10, except when viewing isotopes. Two-peak mobility resolution was calculated as in Dodds *et al.*^37^

### SID device design & operation

For implementation of SID on the G2, a single electrode acting as the surface was fabricated, and the commercial split lens used for DRE functionality was repurposed as extractor and deflector, as described in a recent paper.^22^ For implementation of SID on the cIMS, three stainless-steel SID electrodes were fabricated and installed to replace the DRE lens (Fig. 1b). The extractor and surface electrodes on both instruments are electrically connected, requiring only two voltages (deflector and surface/extractor) for operation. The SID cell on the G2 is powered by the 2 voltages that originally powered the DRE lens (‘collector’ and ‘stopper’ in the instrument software). To make the surface and extractor independent, an external voltage would need to be supplied, but in our testing this was unnecessary. To conduct SID on the G2, the ‘Trap CE’ setting was raised and was matched by the DRE ‘collector’ setting (now repurposed as extractor and surface voltages). Raising the collector setting along with the Trap CE was necessary because in the Synapt G2 configuration the Trap CE also raises the voltages on the DRE lens elements and therefore gives no acceleration prior to the surface unless the collector setting is also raised (thereby dropping the surface voltage). The ‘Stopper’ voltage (now repurposed as the deflector) is set more positive than the surface and extractor in order to cause a surface collision. This configuration has been described previously^22^ and has some limitations, namely that the SID collision voltage is limited to ∼120 V due to internal power supply constraints – the energy range can be extended by using an external power supply and external software – and that the user must manually set the Trap CE, the deflector voltage, and the extractor voltage and optimize the settings for each collision energy. It was thus our goal in this work to wholly integrate SID into the hardware and software of the cIMS.

In contrast, the SID configuration on the cIMS is wholly integrated and simplified to be user friendly. To conduct SID on the cIMS, the user simply types in the SID collision energy as a single setting (analogous to performing CID) and the Waters Quartz instrument control software calculates and applies the correct Trap collision energy and deflector and extractor voltages appropriate for the chosen SID collision energy. For our initial studies in SID mode, the surface and extractor voltages were always kept 10 V above the Trap bias in order to provide some extraction into the Trap after SID. For automation of the deflector and extractor/surface voltages, we chose the following relationship: *V*_D_ = *V*_S/E_ + (1/2)*V*_CE_, where *V*_D_ is the deflector voltage, *V*_S/E_ is the surface and extractor voltage, and *V*_CE_ is the SID collision voltage. In ‘manual’ SID mode the deflector and extractor/surface voltages can be tuned by the user on the cIMS. The three-lens design described here is a notable simplification in the design, operation, and hardware requirements experienced with prior generations of SID devices developed for the Synapt platform in our laboratory^20,38,39^ and, combined with software and hardware improvements on the cIMS represents the first wholly integrated SID system for native mass spectrometry.^22^

## Results & discussion

The performance of the cIMS and the Synapt G2 in terms of both mass and mobility resolution was compared with several model protein complexes that have been used previously to characterize SID performance in different instrument platforms: 53 kDa streptavidin homotetramer, 58 kDa cholera toxin B homopentamer, 43 kDa HFQ65 homohexamer, 115 kDa C-reactive protein, 55 kDa transthyretin tetramer, and 60 kDa neutravidin tetramer, as well as samples of *trp* RNA binding attenuation protein (TRAP) homo-11mer and toyocamycin nitrile hydratase (TNH) heterohexamer labeled with His tags on the *γ* subunits.^40–42^ A set of synthetic aggregates derived from fragments of the amyloid precursor protein^33^ were also characterized by SID-cIMS. We find an overall increase in mobility resolution (median ∼33%) compared to the Synapt G2 but also show that increasing the pathlength further *via* multipass separations around the cIMS array is useful for resolving heterogeneous populations of CIU and SIU conformers.

### SID-cIMS of a homotetramer: mass and mobility resolution

Streptavidin is a *D*_2_-symmetric homotetramer^43^ that serves as a benchmark protein complex for initial SID characterization. Based on interfacial analyses previously conducted,^44^ the protein complex can be described as a dimer of dimers so that low energy SID produces predominantly dimeric species, whereas CID generates highly charged monomer with complementary trimer.

**Fig. 2:**
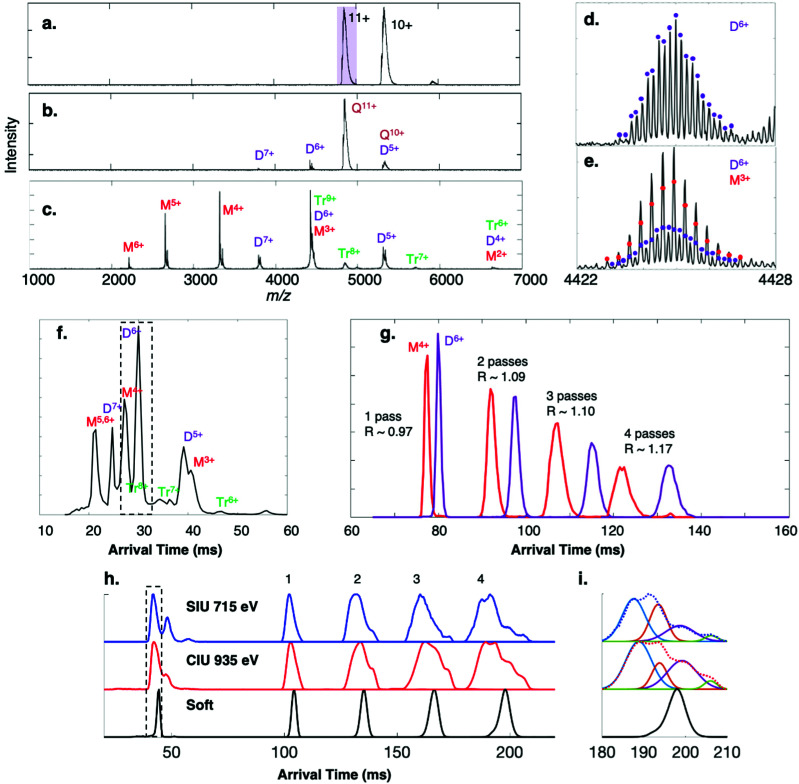
Surface-induced dissociation of 53 kDa homotetramer streptavidin 11+ (charge reduced with TEAA) on the cIMS. (a) Flythrough mass spectrum with purple box indicating ion isolated for MS/MS, and SID spectra at energies of (b) 330 eV and (c) 770 eV and (d and e) corresponding isotope distributions for the peak at *m*/*z* 4424. The mobility distribution of the SID 770 eV spectrum is given in (f). (g) The adjacent dimer 6+ and monomer 4+ species were isolated after a single pass (a ‘slicing’ experiment) and were cycled in the cIMS up to 4 times. At 5 passes the two arrival time distributions overlapped due to the racetrack effect. The 11+ tetramer charge state was subjected to (h) surface-induced unfolding (715 eV) and collision-induced unfolding (935 eV) and the peak corresponding to structural collapse in the dotted box was isolated in the cIMS and passed around the cIMS multiple times, revealing two collapsed conformations and a third conformation with arrival time similar to the native conformation (‘soft’). Panel (i) shows the CIU and SIU 4-pass data fit to four distinct Gaussian distributions and overlaid with the experimentally observed ATD (dotted lines).

Fig. 2a shows the full mass spectrum of streptavidin charge states 9+ through 11+ on the cIMS system, which is comparable to that observed on the Synapt G2 platform (Fig. S1a†). The single pass mobility resolution observed on the cIMS between the 12+ and 11+ charge states was 43% higher than on the Synapt G2 (Table 1). When comparing separation of the 11+ and 10+ charge states, the resolution of the cIMS was 47% higher. Note that two-peak resolution was calculated as per Dodds *et al.*^37^

**Table 1:** Comparison of mobility resolution of native protein complexes and some SID fragments for the cIMS and the G2. Mobility resolution was calculated as in McLean *et al.* (https://pubs.acs.org/doi/full/10.1021/acs.analchem.7b02827). NR = not resolved. M = monomer. D = dimer. T = trimer. Q = tetramer. P = pentamer. H = hexamer. 7 = 7 mer. 8 = 8 mer. 9 = 9 mer. 10 = 10 mer

Protein complex	# passes	Oligomer	cIMS mobility resolution (two-peak)	G2 mobility resolution (two-peak)
Streptavidin tetramers	1 pass	Q	(12+/11+) 1.19; (11+/10+) 1.40	(12+/11+) 0.83; (11+/10+) 0.95
	2 pass	Q	(12+/11+) 1.31; (11+/10+) 1.53	N/A
	3 pass	Q	(12+/11+) 1.35; (11+/10+) N/A	N/A
	4 pass	Q	(12+/11+) 1.36; (11+/10+) N/A	N/A
Neutravidin tetramer	1 pass	Q	(13+/12+) 0.72; (12+/11+) 0.85	N/A
	2 pass	Q	(13+/12+) 0.89; (12+/11+) 0.96	N/A
	3 pass	Q	(13+/12+) N A^−1^; (12+/11+) 1.16	N/A
C-reactive protein pentamer	1 pass	P	(20+/19+) 0.91; (19+/18+) 1.02	N/A
	2 pass	P	(20+/19+) 1.08; (19+/18+) 1.25	N/A
	3 pass	P	(20+/19+) N A^−1^; (19+/18+) 1.32	N/A
Alcohol dehydrogenase tetramer	1 pass	Q	(23+/22+) 0.79; (22+/21+) 0.83	N/A
	2 pass	Q	(23+/22+) 0.91; (22+/21+) 0.97	N/A
	3 pass	Q	(23+/22+) 0.88; (22+/21+) 0.89	N/A
	4 pass	Q	(23+/22+) 0.91; (22+/21+) 0.97	N/A
Streptavidin SID fragments	1 pass	M^3+^/D^6+^/T^9+^	(M/D) 3.2; (D/T) 1.7	(M/D) 2.7; (D/T) 1.5
	1 pass	M/D/T (various charge states)	(M^5,6+^/D^7+)^ 1.3;	(M^5,6+^/D^7+^) 0.8;
			(D^7+^/M^4+^) 1.2;	(D^7+^/M^4+^) 1.0;
			(M^4+^/D^6+^) 0.97;	(M^4+^/D^6+^) 0.47;
			(D^6+^/T^7+^) 2.1;	(D^6+^/T^7+^) NR;
			(T^7+^/D^5+^) 1.0;	(T^7+^/D^5+^) NR;
			(D^5+^/D^6+^) 2.9;	(D^5+^/D^6+^) 2.2;
			(D^5+^/M^3+^) 0.7;	(D^5+^/M^3+^) 0.6;
			(M^3+^/T^6+^) 0.9	(M^3+^/T^6+^) NR
	1 pass	M^4+^/D^6+^	(M^4+^/D^6+^) 0.97	(M^4+^/D^6+^) 0.47
	2 pass	M^4+^/D^6+^	(M^4+^/D^6+^) 1.09	N/A
	3 pass	M^4+^/D^6+^	(M^4+^/D^6+^) 1.10	N/A
	4 pass	M^4+^/D^6+^	(M^4+^/D^6+^) 1.17	N/A
Cholera toxin B SID fragments	1 pass	M^3+^/D^6+^	(M/D) 3.2	(M/D) 2.5
	1 pass	D^4+^/T^6+^/Q^8+^	(D/T) 2.3; (T/Q) 0.9	(D/T) 1.5; (T/Q) 0.3
*holo*TRAP SID fragments	1 pass	M^2+^/D^4+^/T^6+^/Q^8+^	(M/D) 3.0; (D/T) 1.9; (T/Q) 1.2	(M/D) 2.9; (D/T) 1.5; (T/Q) 0.9
	1 pass	Q^4+^/P^5+^/H^6+^/7^7+^/8^8+^/9^9+^/10^10+^	(Q/P) 1.2; (P/H) 1.0; (H/7) 0.9; (7/8) 0.8; (8/9) 0.7; (9/10) 0.6	NR
His-tagged toyocamycin nitrile hydratase (TNH) hexamer and SID fragments	1 pass	(*αβγ*)_2_	(18+/17+) 0.69; (17+/16+) 0.83; (16+/15+) 0.86	(18+/17+) 0.23; (17+/16+) 0.62; (16+/15+) 0.74
	1 pass	(*αβγ*)	(9+/8+) 2.02; (8+/7+) 2.41	(9+/8+) 1.45; (8+/7+) 1.87

The 11+ streptavidin precursor was mass-selected in the quadrupole mass filter of each instrument and dissociated by SID at 330 eV, producing dimer subcomplexes on both the cIMS (Fig. 2b) and G2 (Fig. S1b†) with approximately half the charge of the precursors (∼5.5 charges, on average). Higher-energy SID of the 11+ tetramer at 770 eV on the cIMS (Fig. 2c) and G2 (Fig. S1c†) causes more widespread dissociation into dimer as well as a substantial amount of monomer but only a small amount of trimer (∼1% of the total intensity in each spectrum), consistent with cleavage of the weakest interfaces in this complex.^43^ The improved TOF resolution on the cIMS is evident in panels (d) and (e) as overlapping dimer 6+ and monomer 3+ from low- and high-energy SID are readily resolved even in V mode, with an approximate resolution of 40 000 (FWHM). At the higher SID energy (770 eV) more dimer was observed on the G2 (dimer 7+ and 5+, for example) relative to the monomer. At SID 770 eV, the dimer made up 65% of the total ion intensity on the G2 but only 39% on the cIMS; correspondingly, the monomer made up 27% of the intensity on the G2 but 57% on the cIMS. The decrease in relative abundance of dimer could be attributed to losses along the ion path between the Trap collision cell and the mass analyzer as ions are transferred over additional stages and through an extended path length in the cIMS, though this is only speculative. Even so, the improved cIMS resolution is apparent in the extracted arrival times of the oligomer fragments in Fig. 2f (cIMS) compared to Fig. S1d† (G2, red trace). For example, the adjacent monomer 5+/6+ and dimer 7+ peaks are better resolved on the cIMS. The two-peak mobility resolutions were calculated at *m*/*z* 4424 for overlapping monomer 3+, dimer 6+, and trimer 9+. A two-peak resolution of 3.2 was observed on the cIMS for monomer 3+ and dimer 6+ compared to 2.7 on the Synapt G2, an increase of 18%. For the dimer/trimer pair, the two-peak mobility resolution of 1.7 observed on the cIMS was an approximate 13% improvement from the two-peak separation of 1.5 observed on the G2. For species adjacent to each other in the mobiligram of SID 770 eV, additional two-peak resolution values are given in Table 1. On average, an increase in mobility resolution of approximately 33% was observed on the cIMS (when compared to the Synapt G2) utilizing only a single pass.

A key advantage of using a cIMS instrument is the ability to ‘slice’ and cycle a mobility-selected subset of the ions around the cIMS array multiple times, thereby increasing the mobility resolution.^35,45^ Multipass experiments were conducted with the monomer 4+ and dimer 6+ ions produced from SID 770 eV of streptavidin because, even though they are resolved in the *m*/*z* dimension, they are adjacent in the arrival time dimension and are approximately the same intensity, which is ideal for calculating resolution improvements as the path length is increased *via* multiple passes. The two oligomers were mobility-selected and cycled up to 5 times, with resolution improving from 0.97 at 1 pass to 1.17 at 4 passes (Fig. 1g). At 5 passes (not shown) the monomer 4+ and dimer 6+ overlapped due to the racetrack effect. This modest increase in resolution was also observed when cycling the streptavidin tetramers multiple times, with the resolution of the 12+/11+ pair increasing from 1.19 to 1.31, 1.35, and 1.36 for 1, 2, 3, and 4 passes, respectively. A similar increase in mobility resolution was observed for a variety of other noncovalent protein complexes that were cycled around the cyclic mobility cell up to 4 times. The arrival time distributions of 60 kDa neutravidin tetramers, 115 kDa C-reactive protein pentamers, and 147 kDa alcohol dehydrogenase tetramers were acquired and are shown in Fig. S2.† In general, additional passes improved mobility resolution by approximately 15–25%, as summarized in Table 1. Presumably the theoretical 2× increase in resolution is not observed after 4 passes because of the quasi-continuum of protein conformers that make up even a single ATD in the gas-phase,^45–49^ as observed previously with cytochrome c on the cIMS^45^ and ubiquitin in other IMS studies.^50^ The theoretical 2× increase would only be expected to hold for ‘single’ species with no conformational heterogeneity or flexibility and no ability to interconvert between conformations. Even so, the relative abundances of the monomer and dimer species remained relatively constant, and no unfolding/restructuring (based on peak shape and relative positions of the monomer and dimer) or fragmentation was observed on the ∼140 ms timescale of the ion mobility separation.^35^ Average ion losses of 10% per pass were measured for streptavidin 10+ and 11+ precursors (Fig. S3†), an acceptable loss considering that the racetrack effect is observed at ∼4–5 passes for protein complexes, even when a small mobility slice or single charge state is isolated.

### Extended path length separations reveal overlapping CIU and SIU distributions

Given the notable increase in apparent mobility resolving power observed for most protein complexes produced under “soft” (cool/native) conditions and undergoing multiple cIMS passes, we considered cases in which the conformational heterogeneity of a population of protein complexes may be clarified within only a few passes. Collision-induced unfolding (CIU) and surface-induced unfolding (SIU) experiments proved to be the most promising avenues to explore. The 11+ charge state of streptavidin tetramer was subjected to SIU 715 eV and CIU 935 eV, giving the extracted 11+ ATDs shown below 50 ms in Fig. 2h. Clearly SIU and CIU restructure the tetramer and a heterogenous population of gas-phase ions is produced from both activation techniques. What appears to be a single peak (black dotted box) was “sliced out” of the mobility separation and passed around the cIMS up to 4 times. In both the SIU and CIU experiments, what appears to be one collapsed/compacted conformation after 1 and 2 passes clearly separates into a minimum of 4 conformations after 4 passes, though they are not fully resolved. The SIU and CIU 4-pass data were fit to 4-term Gaussians (with *r*^2^ of 0.9998 for CIU and 0.9984 for SIU) as shown in Fig. 2i. If we compare the SIU and CIU ATDs to the ATDs for native streptavidin 11+ (labeled ‘soft’), then we can clearly see that the two conformations with the lowest ATDs (blue and orange) are collapsed or compacted compared to the native state, whereas the purple conformation appears to have an arrival time only slightly higher than the arrival time measured under ‘soft’ conditions. A reasonable explanation for this peak is that some of the streptavidin 11+ ions remain in a near-native state even after activation. The rightmost peak (green) observed after 4 passes is the same conformation as the middle conformation observed after SIU (or rightmost conformation in CIU), as there was some overlap between the ATDs after SIU and CIU. The resolution of what appears to be a single ATD into four distinct conformations after four passes in both SIU and CIU experiments highlights the utility of multipass experiments coupled to surface and gas-phase collisional activation techniques for probing the conformations of activated protein complexes. We note that we also “sliced” out the front and back portions of the peak in the black dotted box and confirmed that we could change the relative proportions of different conformations, suggesting that we are selecting a mix of conformations and cycling them rather than producing restructured peaks during the cycling. Although we cycled intentionally-activated species here, this approach could also be used for protein complexes that have been “desalted” in the ion source to ensure the declustering conditions did not inadvertently activate the complex of interest, potentially causing multiple restructured isomers to appear.

**Fig. 3:**
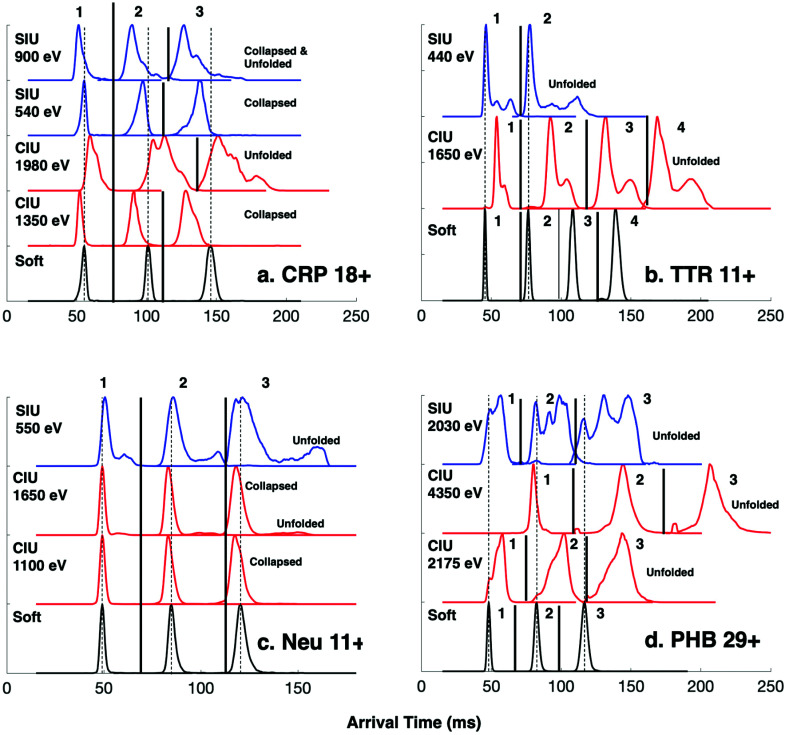
Extended path length separations reveal overlapping CIU and SIU conformations. Comparison of extracted CIU and SIU arrival time distributions to native distributions for the precursors (a) C-reactive protein (CRP) 18+, (b) transthyretin (TTR) 11+, (c) neutravidin (Neu) 11+, and (d) phosphorylase B (PHB) 29+ using up to 4 passes around the cIMS racetrack. Numeric labels correspond to number of passes around the racetrack. Dotted black lines correspond to average arrival time of the native conformation and solid lines separate data traces corresponding to different number of passes.

We conducted similar multipass IMS measurements with 18+ C-reactive protein (CRP), 11+ transthyretin (TTR) tetramer, 11+ neutravidin (Neu) tetramer, and 29+ phosphorylase B (PHB) dimer. CRP, TTR, and PHB have well-studied CIU profiles^51,52^ while SIU and CIU conformations of 11+ neutravidin have been investigated at a single energy.^43^ CRP 18+, for example, has been shown to collapse (6050 *vs.* native 6550 Å^2^) upon modest in-source collisional activation (∼100 V) on a Synapt G2 platform, but then forms expanded conformations (∼7150 Å^2^) at >180 V in-source CIU. Note that here we are collisionally activating in the Trap instead of the source for comparison with surface collisions immediately prior to the Trap. When cycled around the cIMS array three times, the collapsed conformation of CRP 18+ (Fig. 3a, CIU 1350 eV), which appears to be a single ATD after a single pass, partially resolves into two distributions after 3 passes, implying that there are at least two distinct collapsed states. In contrast, when the CIU energy is raised (1980 eV), CRP unfolds or otherwise expands to at least two distributions as observed in 1 pass; this is consistent with two distinct expanded conformers previously detected on a Synapt platform.^51^ However, upon extension of the IMS separation to 2 or 3 passes, at least 3 distinct distributions are observed. The SIU precursor at 540 eV, on the other hand, at first appears to exactly mirror the native precursor with an average arrival time of 55.4 ms and similar width. However, after two and then three passes it becomes clear that even at low SIU energy, a portion of the precursor population is collapsed, though the compaction is not as severe as what is observed with CIU 1350 eV. Even so, *again two distinct collapsed states are observed*. Upon more energetic surface collisions (SIU 900 eV), the CRP precursor collapses further (average arrival time of 51.6 ms in a single pass for the most compact conformer) and after 3 passes begins to resolve into probably 4 distinct states, two of which are collapsed and two which are extended (average arrival times of 127 ms, 135.6 ms, 150.9 ms, and 166.3 ms, *vs.* native arrival time of 146.1 ms after 3 passes).

When subject to CIU at 1650 eV, TTR 11+ clearly restructures to at least two distinct extended/expanded conformations, but upon extension of the path length to 4 passes it becomes apparent that there are at least 3 underlying extended conformations as implied by the shoulder of the more compact conformer (Fig. 3b). Previously, only a single unfolded conformer of the 11+ charge state was detected on a G2 platform using in-source CIU energies of ∼2000 eV (expanded CCS of 3850 *vs.* native 3450 Å^2^).^51^ The SIU (440 eV) precursor unfolds to at least 3 conformations but appears to form different gas-phase structures than were observed by CIU, contrasting with the streptavidin example illustrated in Fig. 2h. The most compact conformer produced by SIU has an average arrival time of 46.3 ms after 1 pass, which is slightly higher than the native conformation (45.4 ms); after 2 passes the arrival times of the SIU conformations are 77.5 ms, 93.4 ms, and 111.6 ms *vs.* 76.6 ms for the native precursor. The two extended conformations agree with our prior work using SIU to characterize *apo* TTR but with the improved mobility resolution and extended pathlength separations it is clear that the most compact SIU conformer is not native but instead slightly expanded.^43^

The collapsed neutravidin 11+ precursors from CIU 1100 eV have a similar behavior to the collapsed CRP distribution in that no clear separation is observed after 3 passes (Fig. 3c). At the two CIU energies tested, the single pass arrival time of the precursor is identical to the native precursor (49.2 ms). However, after 3 passes a clear difference in arrival time is observed (118 ms for the CIU activated conformations *vs.* 120.5 ms for the native state), indicating that although the activated precursors may appear native in a single pass, they do in fact undergo modest compaction that is apparent only after more than 1 pass. The collapsed conformation remains dominant at higher CIU energy (1650 eV) but clearly expanded conformations start to appear, and with 3 passes the conformations partially resolve into 2 expanded/extended distributions. When activated by modest surface collisions at 550 eV, several overlapping unfolded conformations are observed with no clear resolution of the multiple species after 3 passes. The width of the SIU ATD implies that there are multiple underlying conformations, but they were not resolved after 3 passes.

Phosphorylase B (PHB) is a 195 kDa dimer whose CIU and SIU profiles we have published previously.^52^ The 29+ charge state from ammonium acetate was subject to CIU at 2175 and 4350 eV as well as SIU at 2030 eV on the cIMS. PHB 29+ is known to undergo a continuous CIU transition from ∼1500 eV to ∼3500 eV, above which no further restructuring is observed up to 6000 eV. The two CIU energies utilized in the present work (2175 and 4350 eV) were selected to represent complexes which have undergone different extents of restructuring in the gas phase. At the lower CIU energy (2175 eV) a broad distribution of states is observed, consistent with sampling the ‘middle’ of the CIU profile wherein the dimers are transitioning from a well-defined native state to multiple extended/unfolded states (Fig. 3d). Some of the precursor population remains in a native folded state (same ATD as the precursor under soft conditions) while a second broad distribution is observed but not even partially resolved after 3 passes. The broadness of the ATD suggests a great deal of heterogeneity in the underlying conformations. At a higher CIU energy of 4350 eV, the dimer clearly exists in extended/unfolded conformations yet still has a narrow ATD after 1 pass. The width of the peak in the time domain increases much more drastically than the native state after 2 and 3 passes, suggesting that even though the unfolded dimer has a relatively narrow ATD after 1 pass, there is underlying heterogeneity or subunit/complex flexibility (as expected if one or more subunits is unfolded) that exacerbates widening of the peak in the time domain as the IMS path length increases. The unfolding of PHB through surface collisions was studied at a single energy (2030 eV) which produces a heterogeneous distribution of native and restructured dimers. As illustrated by the dotted line, part of the dimer population remains in a native-like state (48.2 ms arrival time after a single pass, 117 ms after 3 passes) but a second prominent peak is observed as well in the single pass data (56.9 ms arrival time). Interestingly, the unfolded dimer resolves into two distinct ATDs (130.6 ms and 148.3 ms) after 3 passes, and neither of the unfolded SIU distributions matches the dominant CIU conformations at either energy, again contrasting with the streptavidin example discussed earlier.

These experiments highlight the ability of longer pathlength separations to benefit CIU and SIU studies of protein complexes. Even though these complexes are considered ‘well-studied standards’, we found that longer pathlength separations revealed new conformations that were not resolved in a single pass and hence improved our understanding of their CIU and SIU conformationomes.^53^

### SID-cIMS of a homopentamer

While multipass separations are clearly beneficial for CIU and SIU experiments of protein assemblies, for SID we found that another very useful feature of the cIMS was the improved single pass mobility resolving power for deconvoluting fragments that overlap in *m*/*z*, which are abundant in SID spectra of cyclic complexes due to symmetric charge partitioning.^54,55^ Cholera toxin B is a homopentamer that has been used to profile the performance of SID-IM,^44^ IM-SID,^56^ and SID with ultrahigh resolution.^22,57,58^ When charge-reduced with TEAA, 10+, 11+, and 12+ pentamers were observed in the mass spectrum on both the cIMS (Fig. S4a†) and G2 (Fig. S5a†). The 11+ pentamer was selected in the quadrupole and fragmented by CID (1980 eV) in the Trap collision cell preceding the IMS on each instrument, producing monomers with 3–4 charges and tetramers with 7–8 charges. The spectra on the cIMS and the G2 are markedly similar. This is a typical result for CID of protein complexes, wherein a monomer is ejected from the precursor complex with a disproportionate amount of charge (4/11 × 100 = 36% of the charge despite the monomer making up only 20% of the total mass of the complex). SID of the 11+ pentamer, on the other hand, at 605 eV on the cIMS (Fig. S4c and d†) and G2 (Fig. S5c and d†) produces monomers, dimers, trimers, and tetramers, with most subcomplexes taking an amount of charge proportional to their mass (*e.g.* dimer taking 4–5 charges on average, commensurate with the theoretical 4.4 charges it should retain under the symmetric charge partitioning model). The improved mobility resolution on the cIMS (compared to the G2) was calculated in Table 1 as approximately 28% for the overlapping monomer 3+ and dimer 6+ and 53% for the dimer 4+ and trimer 6+. Please note that the same color scheme was used to plot both mobiligrams; the difference in absolute color between the plots is due to differences in absolute intensity.

### SID-cIMS of a homo-11mer

**Fig. 4:**
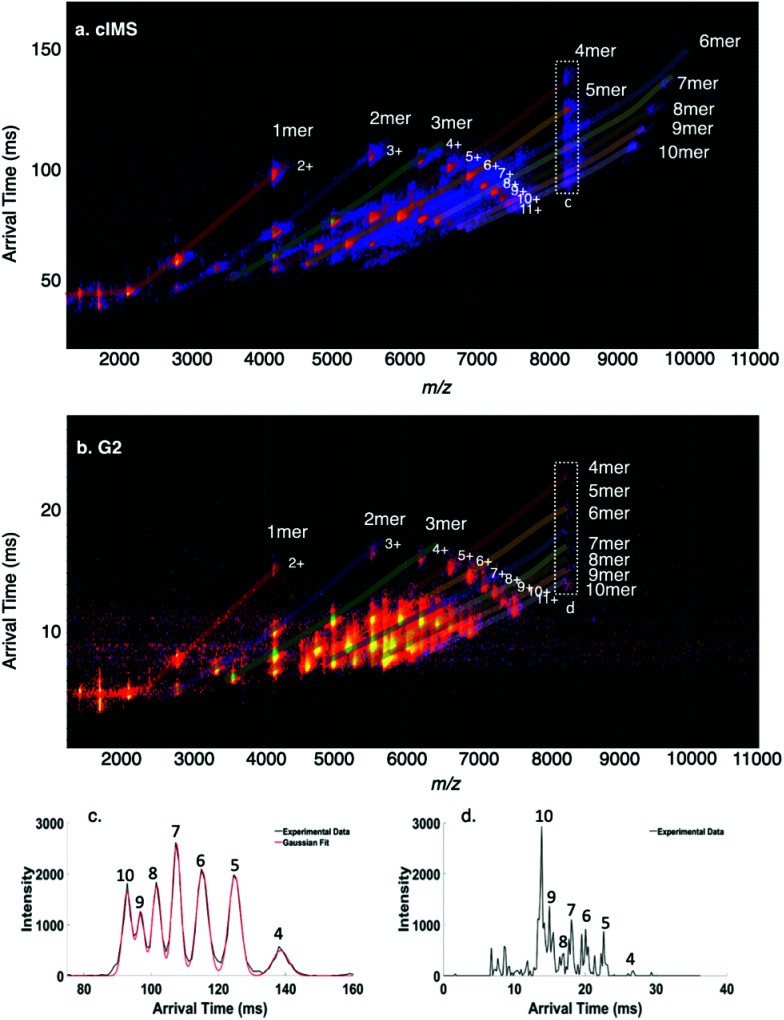
Surface-induced dissociation of 91 kDa homo-11mer *holo*TRAP 16+ (charge reduced with TEAA and with 14 equivalents of *trp*) on the (a) cIMS and (b) G2. The peak at *m*/*z* 8243 consists of at least 7 oligomers which are each evident in the extracted arrival time plots from (c) cIMS and (d) G2 (numeric labels correspond to oligomeric state). Black traces correspond to experimental data and red to Gaussian fits from Matlab.

*Trp* RNA binding attenuation protein, a 91 kDa homo-11mer, has previously been characterized on Synapt,^22,59^ FT-ICR,^22^ and Orbitrap^22,60^ platforms in our laboratory and is more challenging than HFQ65 due to the increased number of oligomers that can be formed by SID. When charge reduced with TEAA, charge states ranging from 15+ to 17+ were detected on the cIMS and Synapt G2. A comparison of the SID-IM spectrum of the mass-selected 16+ 11mer at a collision energy of 1200 eV is shown Fig. 4a (cIMS) and Fig. 4b (Synapt G2). There is a remarkable amount of spectral overlap due to symmetric charge partitioning that is characteristic of SID and the production of many different subcomplexes from the cyclic precursor. The mobility resolution of the overlapping fragments at *m*/*z* 4121 (M^2+^/D^4+^/T^6+^/Q^8+^) was increased by ∼30% on the cIMS compared to the G2, as shown in Table 1. Remarkably, the cIMS was able to not only detect 7 distinct species at *m*/*z* 8243 (4mer through 10mer), but also resolve most of them at half height (excepting 9mer and 10mer) as shown in the extracted ATDs of *m*/*z* 8243 in Fig. 4c, whereas the G2 did not efficiently detect these species (extracted ATD of *m*/*z* 8243 in Fig. 4d) even with the same general settings and acquisition time.

### SID-cIMS of oligomers of Aβ-derived ‘Trimers’

The high mass and mobility resolution of the cIMS platform are not only useful for proteins and protein complexes, but also peptide aggregates. We have recently begun characterizing aggregates of synthetic covalent ‘Trimers’ derived from Aβ_17–36_, which have been pioneered and characterized by the Nowick laboratory.^33,61–63^ A summary of the wide variety of Aβ-derived β-hairpin rich molecules synthesized by the Nowick laboratory is available.^64^ Briefly, the ‘Trimer’ peptides, such as the model molecule Trimer 1_N–Me_ investigated here (shown in Scheme S1†), consist of three β-hairpin peptides derived from Aβ_17–36_ and covalently linked by disulfide bonds. The monomers are antiparallel β strands linked at their N- and C-termini by delta-linked ornithine turn residues (^*δ*^Orn) to form a macrocycle. It was previously discovered that the monomer peptides demonstrate a unique mode of self-assembly into trimers even without disulfide linkages;^65^ the disulfide linkages provide stability to study the higher order aggregates formed from aggregation of the ‘base’ Trimer macrocycles.^33^ As investigation of Aβ aggregation is beyond the scope of the current work, we leave full characterization of these molecules and their derivatives by high-resolution mass spectrometry, cyclic ion mobility, and tandem mass spectrometry to an upcoming manuscript. Here we only wish to utilize aggregates of Trimer 1_N–Me_ to benchmark the capabilities of the SID-cIMS system.

**Fig. 5:**
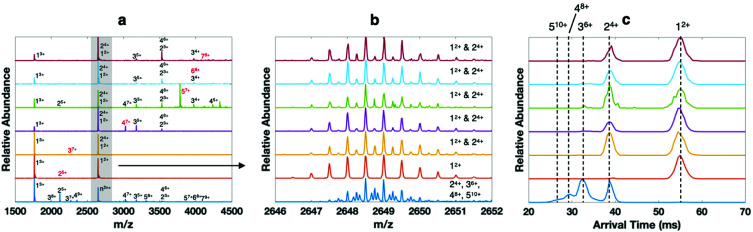
Surface-induced dissociation coupled with high-resolution mass spectrometry and cyclic ion mobility for analysis of homooligomers of Aβ_17–36_-derived ‘Trimer’ 1_N–Me_. (a) native mass spectrum of oligomers from a 40 μM solution of Trimer 1_N–Me_ in 400 mM ammonium acetate (bottom blue trace) and SID spectra of each oligomeric state up to 7mer (the isolated ion subjected to SID is labeled in red, *e.g.*, 2^5+^ (dimer of covalent trimers with 5 charges), 3^7+^, 4^7+^, 5^7+^, 6^8+^, 7^9+^), (b) zoom in of isotopic distributions at *m*/*z* 2649, which can theoretically be any oligomeric state with 2 charges per subunit (n^2*n*+^), and (c) extracted arrival time distributions of *m*/*z* 2649 in each spectrum.

When dissolved at 40 μM monomer concentration in 400 mM ammonium acetate, a variety of oligomers of Trimer 1_N–Me_ are observed on the cIMS, with most of the ion intensity corresponding to 1mer through 4mer but with higher order oligomers clearly present (Fig. 5a, bottom blue trace). Because the homooligomers retain ∼2–3 charges per subunit, on average, and form a continuous distribution of oligomeric states, many peaks in the mass spectrum represent multiple species. For example, *m*/*z* 2649 can theoretically correspond to any size oligomer with two charges per subunit (n^2*n*+^). Based on the isotope distributions in Fig. 5b (dark blue) and cIMS mobility trace in Fig. 5c, *m*/*z* 2649 in the native mass spectrum clearly corresponds to 2mer through 5mer. We selected each size of oligomer from 2mer up to 7mer and conducted SID (Fig. 5a, isolated ions highlighted in red). Again, the peak at *m*/*z* 2649 showcases the utility of the high mass spectral and mobility resolutions (panels b and c, respectively) of the cIMS platform in combination with SID. While the dominant oligomers in the native mass spectrum (bottom trace) are 2mer through 5mer, the SID fragments are clearly only 1mer and 2mer.

### SID-cIMS of a heterohexamer

The final protein we characterized by SID on the cIMS is His-tagged toyocamycin nitrile hydratase (TNH) heterohexamer, which consists of two copies of each of three unique subunits: *α*, 21.2 kDa; *β*, 10 kDa; and *γ*_His_, 11.4 kDa (13.6 kDa with His tag on each copy of *γ*). Blackwell *et al.*^66^ and Song *et al.*^40^ have previously characterized the subunit connectivity of nontagged TNH (11.4 kDa *γ* subunit mass) on an SID-IM-TOF platform and determined that the (*αβγ*)_2_ heterohexamer is a dimer of *αβγ* trimers and that the *αβ* subunit interaction is the strongest within the trimers. Although tagged and nontagged TNH have similar single stage SID patterns,^40,41^ we have observed changes in quaternary structure in SID/SID spectra as evidenced by fragmentation of the *αβγ*_His_ trimer to *α* monomer and *βγ*_His_ dimer on an ultrahigh resolution FT-ICR platform equipped with 2 sequential SID cells (whereas the expected product ions are *αβ* dimer and *γ* monomer). As of this writing, no high-resolution cryo-EM image exists for the complex, though several attempts have been made with limited success.

**Fig. 6:**
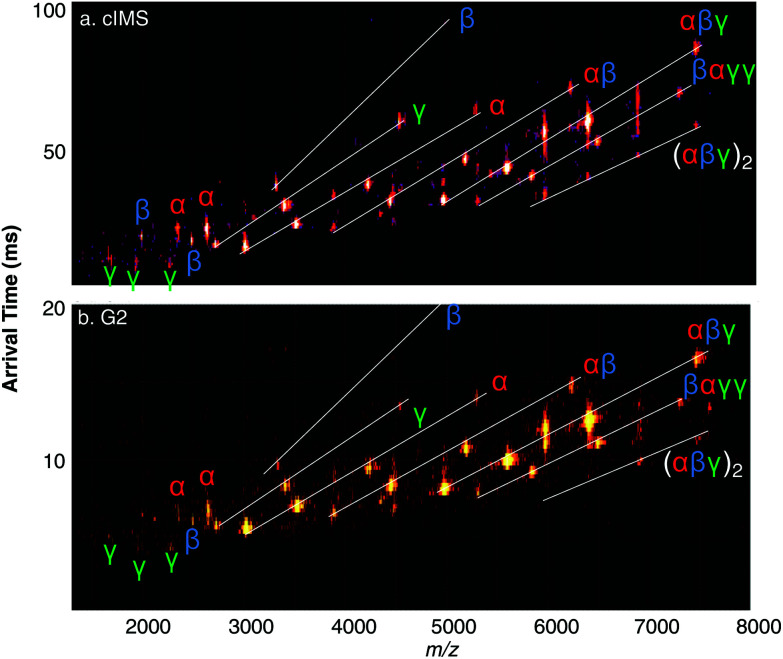
Surface-induced dissociation at 85 V acceleration potential of 89.5 kDa His-tagged (2.2 kDa on each *γ* subunit) heterohexamer toyocamycin nitrile hydratase 15+ on the (a) cIMS and (b) Synapt G2.

Native mass spectra of His-tagged TNH charge reduced with EDDA are shown in Fig. S6† for both the cIMS (left) and Synapt G2 (right). The dominant charge states are slightly different, which may be attributed to source differences (cIMS has a stepwave while the G2 does not) or variations in nanospray tips. The inset extracted ATDs of the 15+ through 18+ precursors (black, 18+; brown, 17+; red, 16+; orange, 15+), which are all normalized to the same intensity, clearly show improved mobility resolution compared to the G2. The 18+ and 17+ charge states, in particular, overlap to a large degree on the G2 but are nearly baseline resolved on the cIMS. The two-peak IMS resolution observed for these hexamers on the cIMS *vs.* G2 are (18+/17+) 2.02 vs 1.45, (17+/16+) 0.83 *vs.* 0.62, and (16+/15+) 0.86 *vs.* 0.74, for an average improvement of 30%. Fig. S6† shows the cIMS and Synapt G2 SID spectra of the His-tagged TNH complex at two different potential differences (45 V and 85 V), with the 15+ charge state (*) selected for fragmentation. At low SID collision energy on the cIMS (SID 45 V, 675 eV), a familiar result is obtained in that the dominant fragments of His-tagged TNH 15+ are *αβγ* trimers. On the cIMS an apparent truncated (∼1.2 kDa lower in mass) protein was observed, even when analyzing the protein directly after buffer exchange. The exact nature of the truncation is unknown. Even so, similar spectra were obtained on the cIMS and G2, with symmetrically distributed heterotrimers as dominant fragments at low SID energy. The inset ATDs (9+ trimer, black; 8+, blue; 7+, green; 6+, purple) again show improved mobility resolution on the cIMS, which is quantified in Table 1. Note that the ATDs marked with * correspond to charge stripped (and likely unfolded) precursors with the same *m*/*z* as the heterotrimer fragments. At higher SID energy (Fig. S6,† bottom, SID 85 *V* = 1275 eV, on cIMS and G2), many additional informative fragments are observed. In both cases all three monomer subunits are observed along with *αβ* dimer since the interface between these subunits is believed to be the strongest.^40^ Both spectra also have minor contributions from *βαγγ* and other unique combinations that are low in relative abundance, which is made more clear in the 2D plots shown in Fig. 6. The improved resolution of the cIMS is evident by visual inspection, particularly since the spectrum is quite congested. Note that we make no claims regarding sensitivity since the G2 platform is much older and has been in use for several years. Overall, the fragmentation patterns are similar, implying that the transmission and SID capabilities of the cIMS are similar to prior Synapt platforms, and native structures are retained throughout the ions’ time-of-flight through the extended ion optics.

## Conclusion

A miniature SID cell using only two voltages has been completely integrated into a cyclic ion mobility mass spectrometer, and the clear benefits of the increased cIMS pathlength of the cIMS system for resolving protein conformations in CIU and SIU experiments as well as overlapping fragments in SID experiments have been demonstrated. The SID cell is wholly integrated into the hardware and software on the cIMS platform, requiring only a single input (SID collision voltage) from the user, an attractive improvement over more complicated SID cells that we have previously developed for native mass spectrometry. The performance of the cIMS in terms of mass and mobility resolution was quantified and compared with a Synapt G2, with substantial increases in TOF resolution allowing isotopic distributions to be observed in cases where multiple oligomers overlapped in *m*/*z*. The isotopic abundances could be verified through improved ion mobility separations, the results of which are highly dependent on the heterogeneity of the quasi-continuum of conformational states that protein complexes and their fragments exhibit in the gas phase.^50^ In some instances we found significant improvements in resolution with, on average, an increase in resolution of ∼33% compared to the Synapt G2. Multiple passes around the cIMS cell improved IM resolution modestly (∼10–20% per pass), and we illustrated several case examples where the extended path length separations partially resolved heterogeneous conformational distributions of complexes restructured by CIU or SIU or where multiple passes were required in order to confirm that CIU/SIU conformations were compacted compared to their native counterparts, despite having identical arrival times after a single pass. The utility of multipass separations is less clear for SID fragments, which are compact and tend to be of low charge and more conformationally homogeneous compared to restructured precursors or highly charged monomers. Overall, this work demonstrates that a completely integrated SID technology and its coupling with high-resolution cyclic ion mobility for native mass spectrometry guided structural biology has significant potential for exploring the topology and conformationome of protein complexes.

## Conflicts of interest

Waters Corporation manufactures and sells SELECT SERIES Cyclic IMS Systems and SID upgrade kits. DTS, BJJ, and VHW are inventors of SID technology being licensed from The Ohio State University by Waters Corporation.

## Supplementary Material

AN-146-D1AN01407B-s001
