# Supplementary material for: Surface-induced dissociation of protein complexes on a cyclic ion mobility spectrometer
Source: Analyst. 2021 Oct 11;146(22):6861–73. doi: 10.1039/d1an01407b (PMC8574189; doi:10.1039/d1an01407b)
Supplement: AN-146-D1AN01407B-s001 [file AN-146-D1AN01407B-s001.pdf]

Electronic Supplementary Material (ESI) for Analyst.
This journal is © The Royal Society of Chemistry 2021

Surface-induced Dissociation of Protein Complexes on a Cyclic Ion Mobility Spectrometer

Dalton T. Snyder‡[1]; Benjamin J. Jones‡[1,2]; Yu-Fu Lin[1,2]; Dale A. Cooper-Shepherd[3]; Darren Hewitt[3]; Jason Wildgoose[3]; Jeffery M. Brown[3]; James I. Langridge[3]; Vicki H. Wysocki*[1,2]

[1]Resource for Native MS Guided Structural Biology
[2]Department of Chemistry and Biochemistry

The Ohio State University, Columbus OH, USA 43210

[3]Waters Corporation, Stamford Avenue, Altrincham Road, Wilmslow SK9 4AX, U.K.

*Corresponding author: wysocki.11@osu.edu
‡These authors contributed equally

Contents

Figure S1 2
Figure S2 3
Figure S3 4
Figure S4 5
Figure S5 6
Figure S6 7
Scheme S1 8
References 9

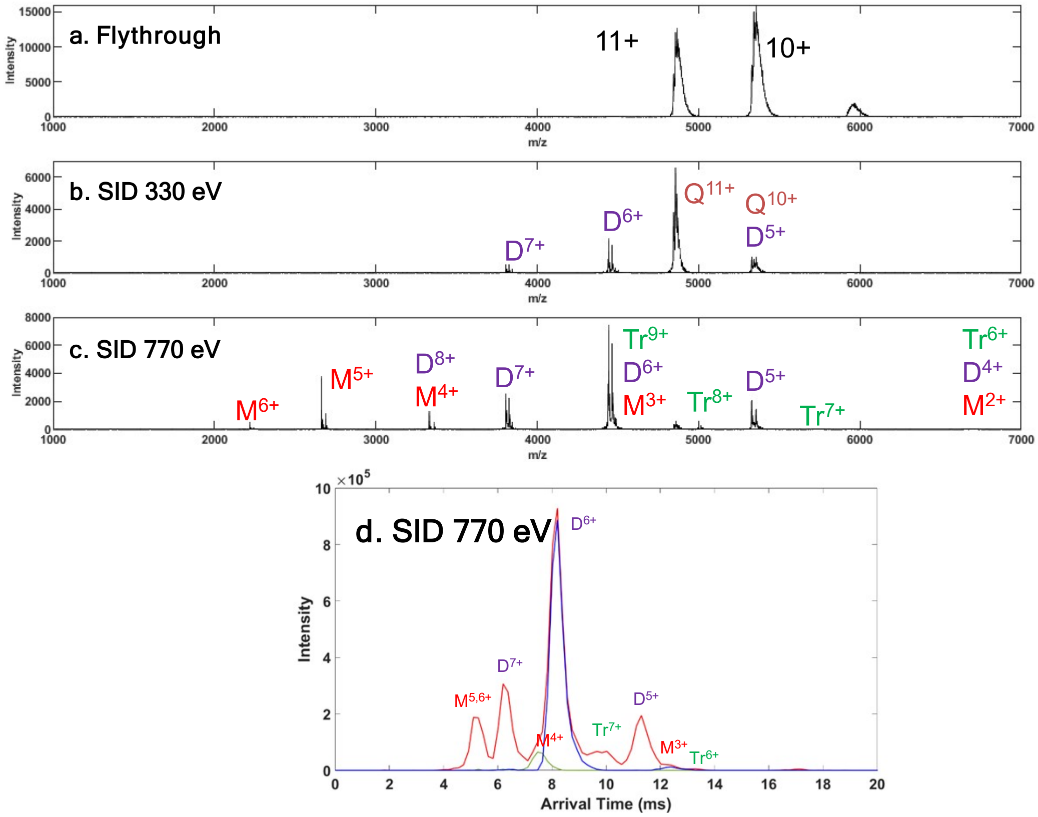

Figure S1: Surface-induced dissociation of 53 kDa homotetramer streptavidin 11+ (charge reduced with TEAA) on a Synapt G2. (a) Flythrough mass spectrum with purple box indicating ion isolated for MS/MS, and SID spectra at energies of (b) 330 eV and (c) 770 eV. Panel (d) shows the extracted arrival times of the ions in panel (c), with the red trace representing all ions, the blue trace representing only D^{6+}, and the green trace representing only M^{4+}.

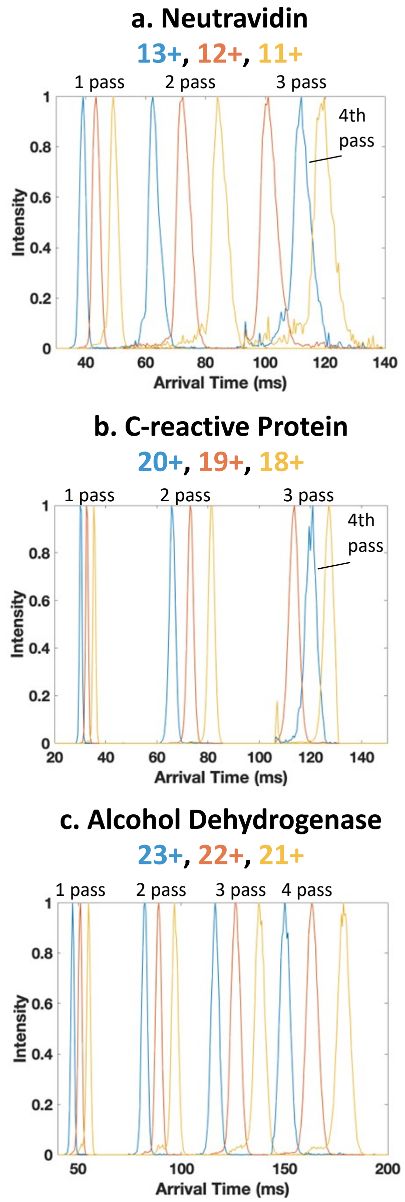

Figure S2: Evaluation of cIMS multipass capabilities for protein complexes (a) neutravidin (60 kDa tetramer, (b) C-reactive protein (115 kDa pentamer), and (c) alcohol dehydrogenase (147 kDa tetramer).

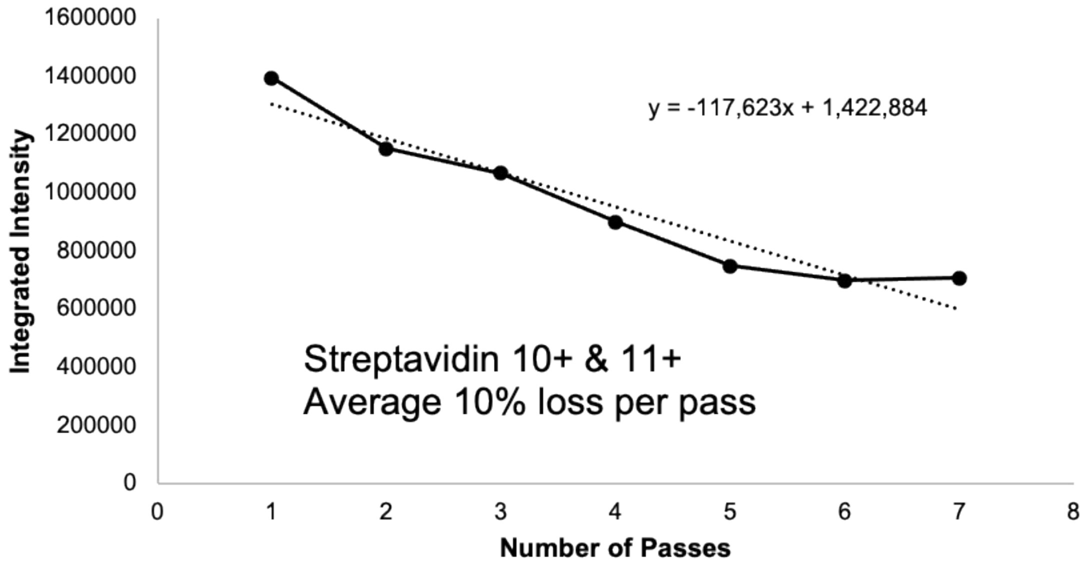

Figure S3: Integrated ion intensity of streptavidin 10+ and 11+ tetramers as a function of number of passes around the cIMS racetrack. On average, 10% loss per pass is observed.

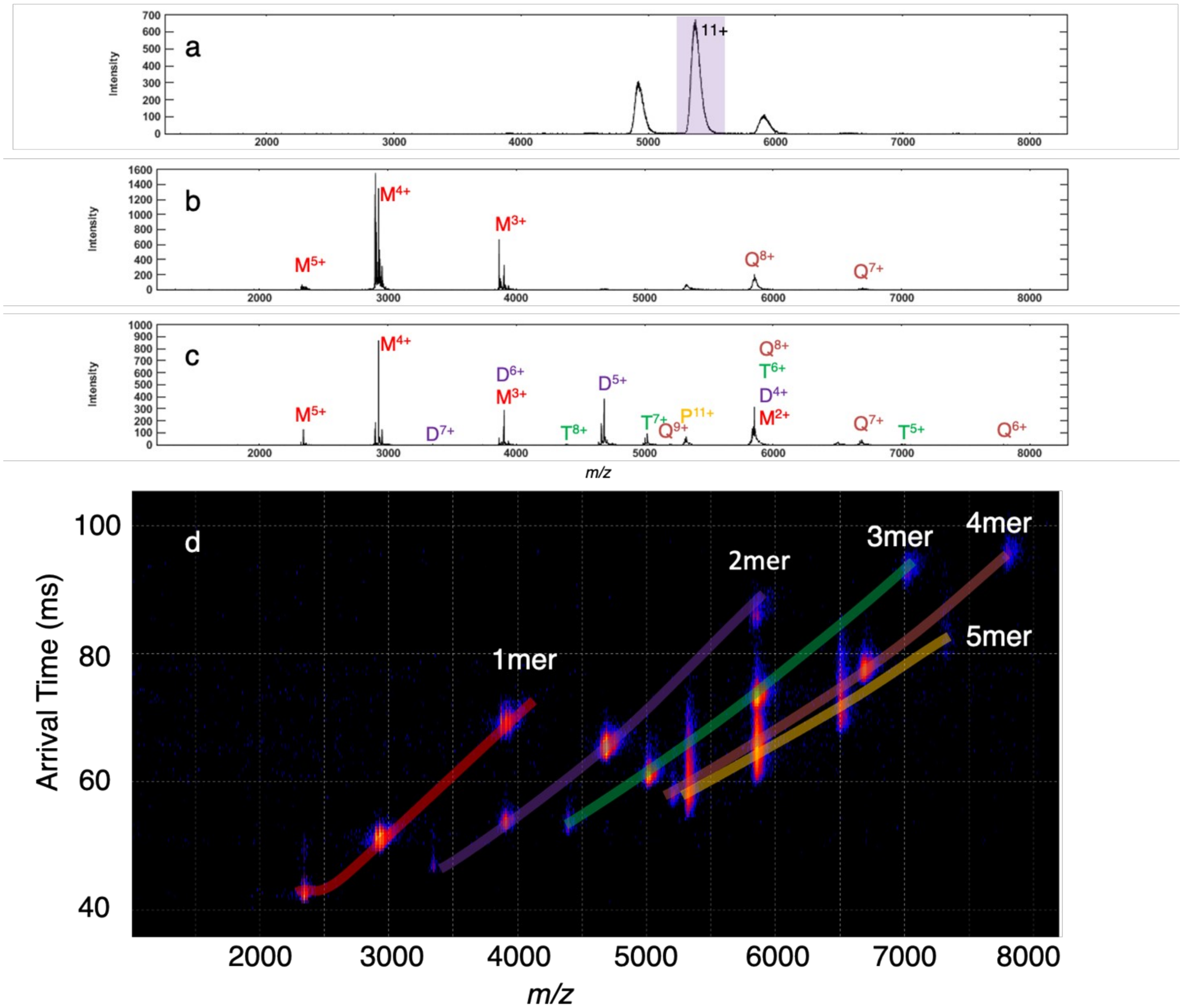

Figure S4: Surface-induced dissociation of 58 kDa homopentamer cholera toxin B 11+ (charge reduced with TEAA) on a cIMS. (a) Flythrough mass spectrum with purple box indicating ion isolated for MS/MS, and (b) CID and (c) SID spectra at energies of (b) 1980 eV and (c) 605 eV. Panel (d) shows the 2D ion mobiligram of the SID spectrum.

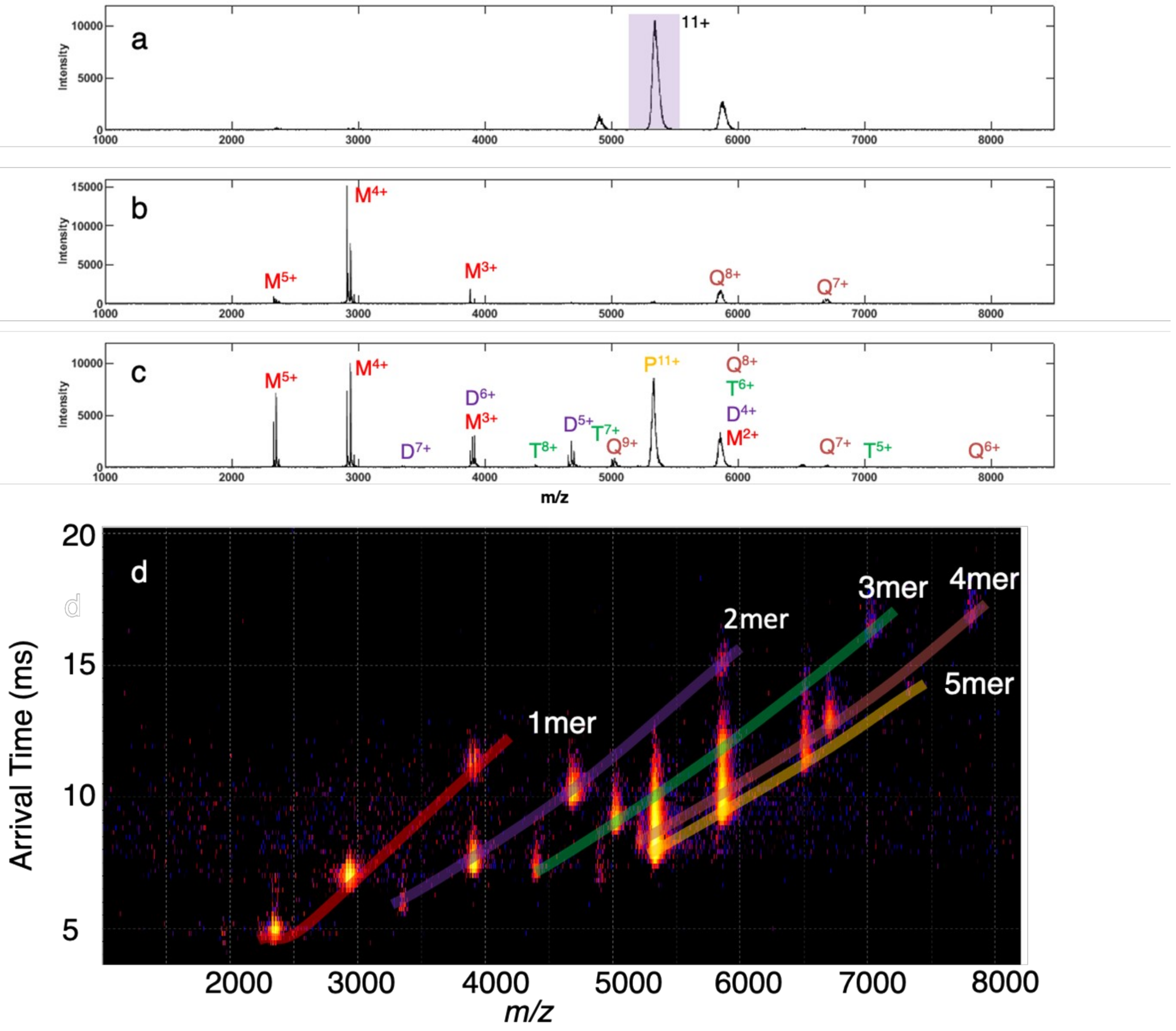

Figure S5: Surface-induced dissociation of 58 kDa homopentamer cholera toxin B 11+ (charge reduced with TEAA) on a Synapt G2. (a) Flythrough mass spectrum with purple box indicating ion isolated for MS/MS, and (b) CID and (c) SID spectra at energies of (b) 1980 eV and (c) 605 eV. Panel (d) shows the 2D ion mobiligram of the SID spectrum.

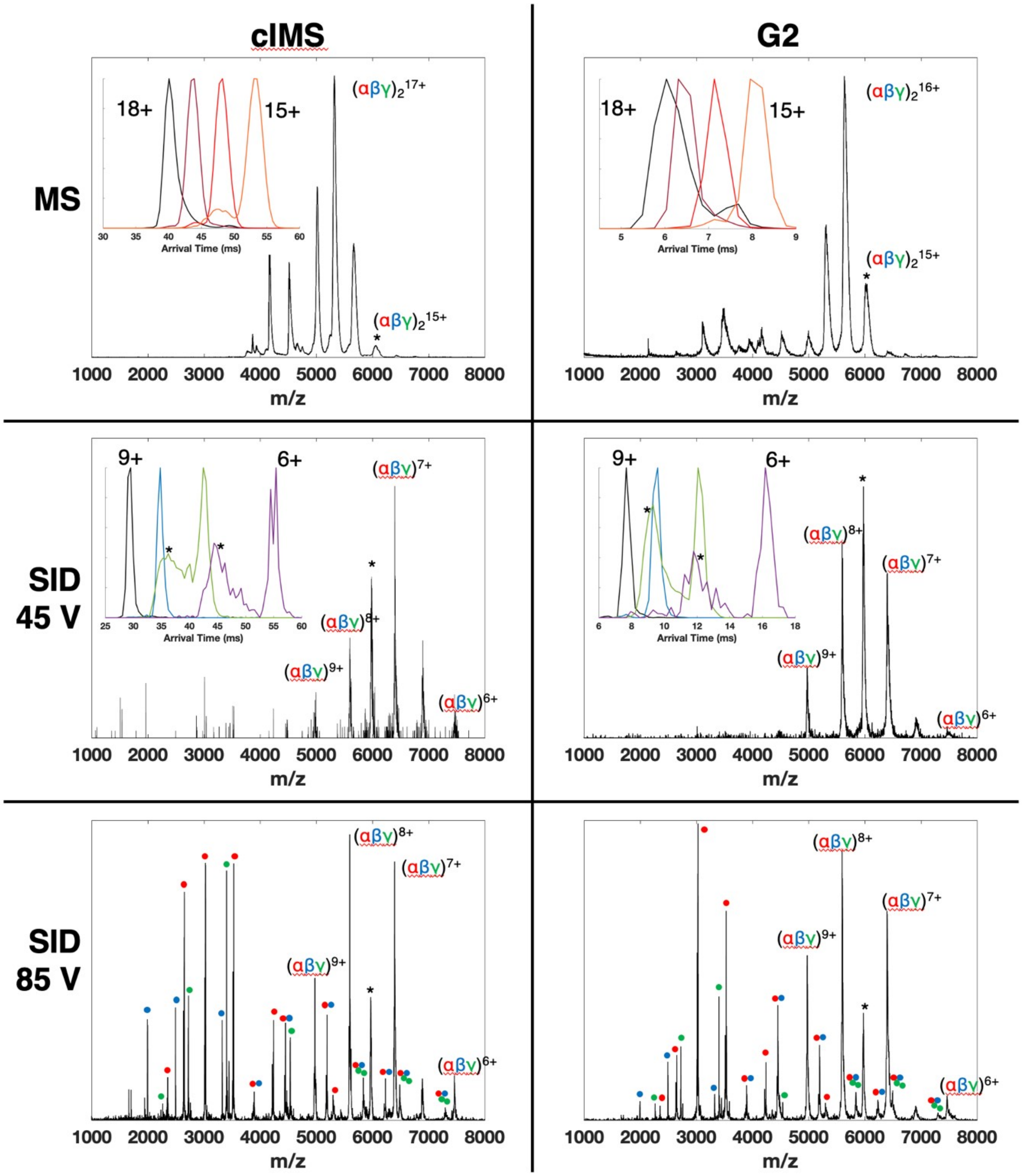

Figure S6: Surface-induced dissociation of 89.5 kDa His-tagged (2.2 kDa on each ɣ subunit) heterohexamer toyocamycin nitrile hydratase 15+ (charge reduced with EDDA) on the (a) cIMS and (b) Synapt G2. The native mass spectra of the charge reduced precursors are shown for reference. Insets show ATDs for (MS) the charge-reduced precursors (black, 18+; brown, 17+; red, 16+; orange, 15+) or (SID 45 V) the αβɣ trimer product ions (black, 9+; blue, 8+; green, 7+; purple, 6+). * corresponds to the precursor ion isolated for MS/MS (or, in the SID 45 V ATDs, surviving 14+ or 12+ heterohexamers from charge-stripping of the 15+ precursor).

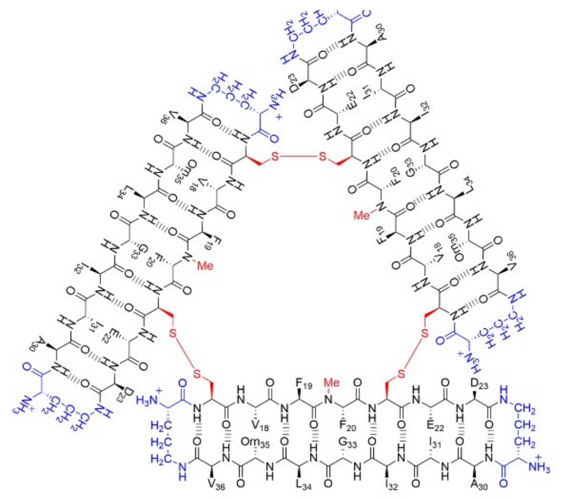

Trimer $1_{N\text{-}Me}$
5295.43 Da

Scheme S1: Structure of Trimer $1_{N\text{-}Me}$ derived from $A\beta_{17\text{-}36}$ [1].

References

1. Kreutzer, A.G., Yoo, S., Spencer, R.K., Nowick, J.S.: Stabilization, Assembly, and Toxicity of Trimers Derived from Aβ. J. Am. Chem. Soc. 139, 966–975 (2017). https://doi.org/10.1021/jacs.6b11748
